# Real-world inter-observer variability of the Sequential Organ Failure Assessment (SOFA) score in intensive care medicine: the time has come for an update

**DOI:** 10.1186/s13054-023-04449-y

**Published:** 2023-04-21

**Authors:** David Pérez-Torres, Pedro Alberto Merino-García, Isabel Canas-Pérez, Cristina Cuenca-Rubio, Guillermo Javier Posadas-Pita, Cristina Colmenero-Calleja, Thalia Gloria Ticona-Espinoza, Cristina Díaz-Rodríguez

**Affiliations:** Servicio de Medicina Intensiva, Hospital Universitario Río Hortega, Gerencia Regional de Salud de Castilla y León (SACYL), Calle Dulzaina, 2, 47012 Valladolid, Spain

**Keywords:** SOFA, Scoring systems, Variability, Reliability, Interobserver agreement

Dear Editor,

Moreno et al. [1] have elegantly highlighted the recent changes in clinical practice and organ support that may not be captured by the SOFA score in its original form, suggesting the need for an update.

Another reason to move to a new version is that the real-world application of the SOFA score in its current form may lack reproducibility. We have empirically noticed that some intensivists strictly follow the original description of the score for calculation, whereas others adopt a more liberal approach, seeking to preserve its original essence in a non-standard fashion. As a result, a patient with acute respiratory failure who is receiving veno-venous extracorporeal membrane oxygenation (VV-ECMO) and has a PaO_2_/FiO_2_ of 190 mmHg may be imputed 3 points (strict approach) or 4 points (liberal approach).

To test this hypothesis, we performed a retrospective study in the ICU of a university hospital in Spain. The study was approved by the Ethics Committee, with a waiver for informed consent. We obtained a random sample by selecting the patients admitted to the ICU at 9:00 a.m. on the 15th of all odd months in 2022. We requested two consultants, two senior and two junior residents to rate the SOFA score from the information available in the electronic medical record. We used the two-way random effects intraclass correlation coefficient (ICC) and the 95% confidence interval (CI) to assess the reliability and consistency of the measurements performed by the different raters. We used the linearly weighted Cohen’s Kappa (κ) and the 95% CI to measure the inter-rater reliability between clinicians with similar professional experience. The ICC or κ values were interpreted as poor (< 0.5), moderate (0.5–0.75), good (0.75–0.9) or excellent (> 0.9) inter-rater agreement [2].

We calculated the SOFA score for 102 patients. The overall ICC (95% CI) of the SOFA score was 0.83 (0.77–0.87). We found the following ICC (95% CI) for the different organ systems: central nervous system (CNS) 0.42 (0.32–0.53), renal 0.62 (0.54–0.70), respiratory 0.65 (0.57–0.72), cardiovascular 0.84 (0.80–0.88), coagulation 0.93 (0.91–0.95), and liver 0.94 (0.92–0.96). The inter-observer agreement according to the degree of professional experience is summarized in Table 1.

**Table 1:** Inter-rater reliability of the SOFA score between clinicians with similar professional experience, expressed as linearly weighted Cohen's Kappa (κ) and the 95% confidence interval (CI)

	Junior residents (2–3 years of experience)	Senior residents (4–5 years of experience)	Consultants (> 6 years of experience)
Central nervous system, κ (95% CI)	0.30 (0.27–0.36)	0.38 (0.31–0.47)	0.64 (0.61–0.73)
Renal, κ (95% CI)	0.59 (0.46–0.78)	0.81 (0.78–0.85)	0.53 (0.46–0.74)
Respiratory, κ (95% CI)	0.68 (0.65–0.80)	0.43 (0.39–0.50)	0.42 (0.40–0.49)
Cardiovascular, κ (95% CI)	0.75 (0.68–0.81)	0.77 (0.76–0.81)	0.86 (0.79–0.89)
Coagulation, κ (95% CI)	0.92 (0.87–0.95)	0.82 (0.74–0.83)	0.98 (0.97–1)
Liver, κ (95% CI)	0.95 (0.92–0.99)	0.82 (0.68–0.88)	0.94 (0.91–0.94)
Overall SOFA, κ (95% CI)	0.61 (0.58–0.66)	0.62 (0.56–0.66)	0.68 (0.61–0.71)

In our study, inter-observer agreement of the overall SOFA score was good. We observed an excellent inter-observer reliability in the liver and the coagulation systems, which can be attributed to the objectivity given by the use of laboratory measurements alone. We found a good inter-rater agreement in the cardiovascular system, where the small differences detected may be explained by the use of inotropes like dobutamine or levosimendan, or mechanical circulatory support, which are not captured by the original SOFA score. We identified a moderate inter-observer agreement in the evaluation of the respiratory and renal systems. The moderate agreement in the respiratory system may reflect the wide range of respiratory support devices available nowadays, which include high-flow oxygen therapy, non-invasive and invasive ventilation, or VV-ECMO. On the other side, the original score does not take into account the possibility of using SpO_2_ when arterial blood gas analysis is not available. The variability detected in the renal system is explained by the use of renal replacement therapy (RRT) or the removal of urinary catheters to reduce the risk of infection. Finally, we observed a poor agreement in the assessment of the CNS, which we attribute to the inherent subjectivity in the evaluation of the Glasgow Coma Scale, particularly in patients under sedation or mechanical ventilation. When considering the possibility that professional experience may influence the reliability of the SOFA score, our data demonstrates that overall agreement between clinicians with the same level of expertise remains only moderate.

Previous studies have described a good inter-observer agreement for the overall SOFA score. Arts DGT et al. [3] found an ICC of 0.89 in 2005, with weighted κ coefficients similar to the ones we obtained for the hepatic and coagulation systems (> 0.9) and the circulatory system (0.75–0.9). However, the reported κ coefficients for the renal (0.85), respiratory (0.63), and CNS (0.55) were higher than the ones we observed. We consider the downtrend in the agreement in these organ systems over time can be explained by the wider availability of RRT and respiratory support devices, as well as the implementation of light sedation policies and screening tools for delirium. Yet, another study by Tallgren M et al. [4] pointed out that less than half of the SOFA scores calculated by physicians were accurate in real-world practice. Training, education, and comprehensive guidelines may improve the accuracy of the SOFA score [4, 5].

We believe that the SOFA score should be updated to reflect the trends in current clinical practice and organ support, and that this should be complemented by a training program to increase its accuracy and minimize inter-observer variability.

## Data Availability

The datasets used and/or analyzed during the current study are available from the corresponding author on reasonable request.

## Abbreviations

SOFA: Sequential Organ Failure Assessment
VV-ECMO: Veno-venous extracorporeal membrane oxygenation
PaO_2_: Partial pressure of oxygen
FiO_2_: Fraction of inspired oxygen
ICU: Intensive care unit
ICC: Intraclass correlation coefficient
CI: Confidence interval
κ: Cohen’s Kappa
CNS: Central nervous system
SpO_2_: Peripheral oxygen saturation
RRT: Renal replacement therapy
